# Editorial: Error-related potentials: Challenges and applications

**DOI:** 10.3389/fnhum.2022.984254

**Published:** 2022-07-19

**Authors:** Gabriel Pires, Miguel Castelo-Branco, Christoph Guger, Giulia Cisotto

**Affiliations:** ^1^Institute of Systems and Robotics, University of Coimbra, Coimbra, Portugal; ^2^Department of Engineering, Polytechnic Institute of Tomar, Tomar, Portugal; ^3^Coimbra Institute for Biomedical Imaging and Translational Research, Coimbra, Portugal; ^4^Faculty of Medicine, University of Coimbra, Coimbra, Portugal; ^5^g.tec medical engineering GmbH, Schiedlberg, Austria; ^6^Department of Informatics, University of Milan-Bicocca, Milan, Italy

**Keywords:** error-related potentials, Nc/CRN, Ne/ERN, ACC, insula, theta rhythms

Error-related potentials (ErrPs) are neurophysiological signals associated with error processing. They are generated when wrong actions are perceived and have been reported in many contexts in the past three decades, namely when a subject perceives that he/she has committed an error and recognizes it immediately in choice reaction time paradigms (“response ErrP”), when a subject receives the feedback of a previous choice without knowing whether it was wrong (“feedback ErrP”), when observing mistakes of another person or an intelligent agent (“observation ErrP”) or during the interaction with a Brain-Computer Interface (BCI) when the feedback is not the expected one (“interaction ErrP”). The components of an ErrP appear within a time window of 500 ms and are naturally elicited in the brain without the user's explicit intention. Thus, its automatic detection can be used in real-time in myriad ways. Given the importance of error monitoring in social interaction, behavior, human-machine interaction, and cognitive learning, it starts to be recognized that the possibility of automatic detection of error signals through machine learning can be relevant for many real-life applications in clinical and non-clinical contexts. ErrPs have already been applied as a proof-of-concept in several applications, for detection and correction of BCI choices to increase reliability, to adapt BCI systems over time, or to make artificial intelligent systems learn. Furthermore, in recent years there has been a growing interest in the integration of ErrP-based approaches in clinical applications for disorders where error monitoring is impaired. While the practical use of error signals is still in its infancy and is an open research field, there is also much to know in order to understand their origin and underlying neural mechanisms.

Aiming at contributing to this research field, the special Research Topic on challenges and applications of ErrPs was launched in Frontiers in Human Neuroscience – Brain-Computer Interfaces, which brought together inputs from clinical and basic neuroscience, psychology, and engineering, presenting new neurophysiological insights about error signals, novel applications, both in terms of original contributions and literature reviews.

ErrPs are typically characterized by a midfrontal Error Negativity (Ne) also called Error-related Negativity (ERN) appearing after errors have been committed, which is followed by a centro-parietal Positive deflection (Pe) reflecting conscious recognition of error. Additionally, it has been observed that correct trials also elicit an Error Negativity-like wave called Correct-Related Negativity (CRN or Nc). Functional magnetic resonance imaging (fMRI) studies show that the source of ERN and CRN is the anterior cingulate cortex (ACC). Whether ERN and CRN are part of the same component has been a matter of debate. Vidal et al. examine the similarities and the differences existing between the Ne and the Nc regarding their functional properties and their anatomical origin. This analysis is of utmost relevance as if Nc is considered a small Ne then it questions the existing models. Vidal et al. systematically review and analyse several theories, presenting an unbiased perspective about their possible validity, and conclude that Ne and Nc share a main common generator.

Besides ErrPs, midfrontal theta rhythms have shown to be a relevant biomarker in error monitoring processes. Estiveira et al. studied how performance monitoring is reflected by midfrontal theta rhythms, during response preparation and execution, and whether midfrontal theta depends on the performed action. The authors found an increase in theta power during error commission while there was a lower theta power preceding error responses when compared to correct responses. These results indicate that theta rhythms could be used to improve error detection in BCIs as well as help anticipate the occurrence of errors. The study of Estiveira et al. also found that the midfrontal theta was independent of the type of task, which suggests that these results can be generalized to other tasks including BCI.

Castelhano et al. conducted a study involving a deep computer programming source-code comprehension task that included error monitoring to detect bugs in the code. The authors wanted to validate the hypothesis that the insula is involved in deep error monitoring. Importantly, a strong causal top-down effect from the anterior cingulate cortex to the insula was observed in the presence of errors. This region is pivotal in error monitoring and the insula relates to task difficulty and performance.

BCI spellers are one of the applications that has aroused the most interest in the BCI community. There have been many ways to improve the reliability and communication rate of these devices. Despite many advances, there is still much room for improvement. The incorporation of error-related potentials is one of the ways to improve system performance. As a new contribution, Gonzalez-Navarro et al. used ErrPs to improve the reliability of a speller based on a rapid serial visual presentation (RSVP) paradigm. The system fuses the information coming from event-related potentials (ERPs), ErrPs and context information (language model) in a Bayesian framework, showing to improve speller speed and accuracy.

The idea of using ErrPs as a reward to make intelligent systems learn or adapt through reinforcement learning has raised a lot of interest from the BCI community in recent years. Fidêncio et al. review existing literature, in which ErrPs are used as a reward in reinforcement learning-based setups. The review presents and categorizes the main investigated approaches, and most importantly discusses the strengths and weaknesses of such strategies, pointing out aspects that can be improved.

Despite the remarkable and successful use of ErrPs and other error-related neural signatures, it is clear that there are many challenges to overcome to get the most out of error recognition, including low accuracy of single-trial detection, calibration requirement, low generalization across applications/tasks/subjects and asynchronous detection in continuous tasks. These challenges have been dictating the use of automatic error recognition mainly for highly controlled (sometimes unrealistic) applications/scenarios. Overall, this Research Topic contributed to important theoretical and practical achievements and to highlighting new potential uses of error signals, which will certainly serve to stimulate further research in this promising field.

## Author contributions

GP and MC-B wrote the first draft of the manuscript. GP, MC-B, CG, and GC provided critical revision of the manuscript and important intellectual contributions. All authors read and approved the submitted version.

## Funding

This work has been financially supported by Portuguese Foundation For Science and Technology (FCT) under grants B-RELIABLE: PTDC/EEIAUT/30935/2017 and BCI-CONNECT: PTDC/PSIGER/30852/2017.

## Conflict of interest

CG is the owner of g.tec medical engineering GmbH. The remaining authors declare that the research was conducted in the absence of any commercial or financial relationships that could be construed as a potential conflict of interest.

## Publisher's note

All claims expressed in this article are solely those of the authors and do not necessarily represent those of their affiliated organizations, or those of the publisher, the editors and the reviewers. Any product that may be evaluated in this article, or claim that may be made by its manufacturer, is not guaranteed or endorsed by the publisher.

